# Development and internal validation of a dynamic fall risk prediction and monitoring tool in aged care using routinely collected electronic health data: a landmarking approach

**DOI:** 10.1093/jamia/ocae058

**Published:** 2024-03-26

**Authors:** Nasir Wabe, Isabelle Meulenbroeks, Guogui Huang, Sandun Malpriya Silva, Leonard C Gray, Jacqueline C T Close, Stephen Lord, Johanna I Westbrook

**Affiliations:** Centre for Health Systems and Safety Research, Australian Institute of Health Innovation, Macquarie University, North Ryde, NSW 2109, Australia; Centre for Health Systems and Safety Research, Australian Institute of Health Innovation, Macquarie University, North Ryde, NSW 2109, Australia; Centre for Health Systems and Safety Research, Australian Institute of Health Innovation, Macquarie University, North Ryde, NSW 2109, Australia; Centre for Health Systems and Safety Research, Australian Institute of Health Innovation, Macquarie University, North Ryde, NSW 2109, Australia; Centre for Health Service Research, Faculty of Medicine, The University of Queensland, Brisbane, QLD 4072, Australia; Neuroscience Research Australia, University of New South Wales, Sydney, NSW 2052, Australia; School of Clinical Medicine, University of New South Wales, Sydney, NSW 2052, Australia; Neuroscience Research Australia, University of New South Wales, Sydney, NSW 2052, Australia; School of Population Health, University of New South Wales, Sydney, NSW 2052, Australia; Centre for Health Systems and Safety Research, Australian Institute of Health Innovation, Macquarie University, North Ryde, NSW 2109, Australia

**Keywords:** falls, residential aged care, nursing homes, fall risk prediction

## Abstract

**Objectives:**

Falls pose a significant challenge in residential aged care facilities (RACFs). Existing falls prediction tools perform poorly and fail to capture evolving risk factors. We aimed to develop and internally validate dynamic fall risk prediction models and create point-based scoring systems for residents with and without dementia.

**Materials and methods:**

A longitudinal cohort study using electronic data from 27 RACFs in Sydney, Australia. The study included 5492 permanent residents, with a 70%-30% split for training and validation. The outcome measure was the incidence of falls. We tracked residents for 60 months, using monthly landmarks with 1-month prediction windows. We employed landmarking dynamic prediction for model development, a time-dependent area under receiver operating characteristics curve (AUROCC) for model evaluations, and a regression coefficient approach to create point-based scoring systems.

**Results:**

The model identified 15 independent predictors of falls in dementia and 12 in nondementia cohorts. Falls history was the key predictor of subsequent falls in both dementia (HR 4.75, 95% CI, 4.45-5.06) and nondementia cohorts (HR 4.20, 95% CI, 3.87-4.57). The AUROCC across landmarks ranged from 0.67 to 0.87 for dementia and from 0.66 to 0.86 for nondementia cohorts but generally remained between 0.75 and 0.85 in both cohorts. The total point risk score ranged from −2 to 57 for dementia and 0 to 52 for nondementia cohorts.

**Discussion:**

Our novel risk prediction models and scoring systems provide timely person-centered information for continuous monitoring of fall risk in RACFs.

**Conclusion:**

Embedding these tools within electronic health records could facilitate the implementation of targeted proactive interventions to prevent falls.

## Introduction

Falls represent a significant public health concern.^1–4^ They are common in older adults in residential aged care facilities (RACFs), surpassing the incidence in the community by >3-fold.^2^^,^^5^ They are a leading cause of hospitalization for RACF residents^6^ underscoring their significance as a vital indicator of safety and quality of life in this setting.^7^ Recognizing their significance, it is important to design and implement effective fall prevention programs.^8–13^ Fall prediction is the first step in fall prevention programs. Appropriate fall prediction can help identify older adults at high risk of falling and alert older adults, caregivers, and health administrators before the occurrence of a fall.^11^ Precise fall prediction can also contribute to tailored interventions to reduce fall risk and improve outcomes, as well as the containment of medical and health care expenditure related to falls.^1^^,^^14^ With ageing populations in many countries, the anticipated rise in fall-related injuries and costs underscores the importance of this issue.^15^

Fall prediction is complex due to the multiplicity of factors,^16–18^ including medical, medication, functional, behavioral, physiological, and environmental factors,^16^^,^^19^ many of which (eg, medication use) may change over time.^11^ Existing fall risk assessment tools rely on data collected at a single time point without using day-to-day modifiable risk factors (eg, medication administered), exhibit poor predictive performance, and are unable to capture the dynamic nature of fall risk or reflect changes in risk profile over time.^8^^,^^16^^,^^17^^,^^21–25^ Commonly used tools such as the Peninsula Health Falls Risk Assessment Tool (PH-FRAT) are limited by such problems, hindering their usefulness and validity in fall prediction as a result of the outdated data on which they are based.^17^^,^^18^^,^^21^^,^^26^^,^^27^ Additionally, recent international guidelines recommend multifactorial fall risk assessments for every RACF residents upon their day of admission and repeated annually afterward.^20^ However, this recommendation might not be practical for many RACFs given its labor-intensive nature and the understaffing reality of the aged care sector. An effective fall prediction tool that can automatically and dynamically identify RACF residents with urgent falls intervention needs can make up such deficiency.

Dynamic prediction models overcome limitations by offering real-time risk predictions,^28–30^ integrating time-varying and fixed factors, and allowing for targeted response to changing information.^31^ Despite prior use in other contexts,^31–34^ dynamic prediction models have yet to be applied to fall prediction in RACFs. The increasing availability of routinely collected electronic data in RACFs provides an opportunity for applying dynamic prediction models for fall prediction.^35^ Routinely collected aged care data, obtained through daily care using electronic information systems, hold substantial potential for enhancing care delivery and optimizing resource allocation.^35^ This study aimed to: (1) develop and internally validate dynamic fall risk prediction tool using a landmarking dynamic prediction approach; and (2) to create point-based scoring systems for determining the risk of falls. This study is part of a larger program of work to develop and test a predictive dashboard to improve aged care.^36^

## Methodology

### Setting and design

A retrospective longitudinal cohort study was conducted using over 8 years of electronic data (July 2014-August 2022) extracted from 27 RACFs in Sydney metropolitan area, New South Wales (NSW), Australia. This study was reviewed and approved by the NSW Population and Health Services Research Ethics Committee (Ref: 2020/ETH00166) and the Macquarie University Human Research Ethics Committee (Ref: 52019614412614). We followed the REporting of studies Conducting using Observational Routinely-collected health Data (RECORD) guidelines to report results.^37^

### Participants

The eligibility criteria included permanent residents aged ≥65 years, and those who stayed in the facilities for ≥30 days to complete at least the first prediction window. We limited our analyses to residents who received at least one assessment using the Peninsula Health Falls Risk Assessment Tool (PH-FRAT),^38^ as some variables for the study will be extracted from these assessments. The PH-FRAT is a widely used tool in Australian RACFs with 9 out of 10 residents receiving an assessment during their stay.^39^ Brieftly, the PH-FRAT comprises 3 components: categorizing residents’ fall risk levels, evaluating the risk factors of falls, and documenting the fall prevention intervention strategies required (see https://training.aacs.com.au/wp-content/uploads/2016/08/Falls-Risk-Assessment-Tool-FRAT.pdf).

### Data source and candidate predictors

We linked 4 aged care databases (resident profile, medication administration, PH-FRAT, and incident reports) to obtain comprehensive data relevant to this article (Table S1). The *resident profile* contains demographics (eg, age and sex) and a free-text column that contains information pertaining to 70 health conditions (eg, dementia and diabetes). We conducted a literature search to identify conditions associated with an increased risk of falls, excluding those with a prevalence of <5%. This led to the selection of 20 conditions as candidate predictors. All variables sourced from the *resident profile* were time-invariant and recorded only at baseline.

Medication administration contains data on medications each resident received daily. We extracted 36 variables from this dataset, including 13 related to fall-risk increasing drugs (FRIDs), 21 related to other medication classes, and variables indicating polypharmacy (≥9 regular medications) and sedative load. FRIDs include medications (eg, antipsychotics) that increase fall risk through their effects on the central nervous system and medications (eg, beta blockers) that increase fall risk by causing orthostatic hypotension.^41^^,^^42^ We used the Anatomical Therapeutical Chemical (ATC) classification codes to identify relevant medications. The ATC classification system includes 5 levels which categorize active substances into groups based on their effects on specific organs or systems by considering their therapeutic, pharmacological, and chemical properties.^43^ Sedative load is calculated by summing the sedative ratings of medications based on their potential to cause sedation with a score of ≥3 indicating a high sedative load.^44^ All medication-related variables are time-varying and can potentially change daily.

The *PH-FRAT database* contains data on the existing fall risk assessment tool (ie, PH-FRAT). Details about the nature of data collected and the performance of the PH-FRAT have been described elsewhere.^39^ We utilized 6 variables from this database (Table S1). All PH-FRAT variables were time-varying with their value updated every time a new PH-FRAT assessment was conducted. The *Incident* database contains data on all falls incidents including date and time of incidents. The incidence of falls in the study cohort was reported previously using the same database.^45^

### Outcome measure

The outcome measure was the incidence of falls. That is, all falls were included regardless of whether they led to injury or transferred to hospital for further evaluation and/or management. The study has considered all reported falls events in the database, encompassing information of falls incidents reported by staff/nurses, and those self-reported by residents and recorded by staff/nurses.

### Missing data

There were no missing data for variables sourced from the *resident profile*, *incident reports*, and *medication administration* database. We did not consider a few variables with significant missing data in these 3 datasets even though they might affect fall risk. For the 6 variables obtained from the PH-FRAT database, there were minimal instances of missing values (<1% for certain variables at specific time points during follow-up and observed they were not systematically missing). To address this, we applied the *last observation carried forward* approach for a variable value missing during the follow-up period and the *next observation carried backward* approach for a variable value missing at earlier time points.

### Sample size

We included all participants meeting the described criteria, enrolling 5492 participants. Over three-quarters experienced at least one fall during the study period, yielding a substantial number of events per predictor parameter, exceeding the minimum required value^46^ and mitigating model overfitting.

### Statistical analysis

#### Dynamic fall risk prediction

The FRIPAC tool has 2 main components namely a predictive model and a monitoring tool. We utilized the landmarking approach to develop a dynamic fall risk predictive model. The landmarking approach, initially introduced by van Houwelingen,^47^ requires few modeling assumptions, is robust against mis-specifications, and is easily implemented using standard software.^48^ Given falls are a recurrent event, we applied the extension of landmarking approach to a recurrent event outcome scenario, adopting similar techniques proposed previously.^49^^,^^50^

Landmarking requires the creation of a super prediction dataset. A detailed description of how to create a landmark dataset and modeling options in the setting of recurrent event outcomes has been described by Musoro et al^49^ and Liu.^50^ We tracked residents for 60 months, using monthly landmarks with 1-month prediction windows. This approach involves creating a large super prediction dataset, as outlined in the Box S1. We applied a *stratified Cox landmark supermodel*, an extended Cox model, to dynamically estimate fall probabilities at each landmark, using robust standard errors to account for potential multiple appearances of the same residents in the dataset. In the setting of recurrent event outcomes, it is critical to utilize an observed event history as a predictor of future events.^49^ In this study, we used recent falls history (falls in the last 6 months prior to a landmark) as a time-dependent predictor of risk of falls in the subsequent landmark.

#### Model development and internal validation

We developed separate models based on dementia status. We randomly split the super prediction dataset into training and validation samples in a 70:30 ratio, respectively. We used the Collett’s approach for variable selection (Box S2).^51^

#### Model performance measures

The final model performance was determined using the validation sample. We used dynamic area under receiver operating characteristics curve (AUROCC), a time-dependent (landmark-specific) measure of how well the model correctly differentiates between residents with or without a fall in each landmark. This dynamic AUROCC estimation was based on the approach described by Heagerty et al.^52^ The AUC values from 0.5 to 0.6, 0.6 to 0.7, 0.7 to 0.8, 0.8 to 0.9, and 0.9 to 1.0 indicate “poor,” “sufficient,” “good,” “very good,” and “excellent” discrimination, respectively.^53^ We also reported dynamic sensitivity and specificity values of the model.

#### Full model specifications, point-based scoring system, and risk staging

The training and validation samples were combined to fit the full dynamic prediction model. We adopted a regression coefficient-based approach to develop a point-based scoring system to develop the FRIPAC monitoring component.^54^^,^^55^ The total score can be a negative value (indicating a protective effect), zero (signifying no risk), or a positive value (suggesting a risk factor). One commonly used variant of this approach, which we implemented, multiplies the regression coefficients by a scaling factor of 10 and rounds the values to the nearest integer.^55^ Four fall risk stages were determined using the 16th, 50th, and 84th centiles of the prognostic index (PI), a weighted sum of the variables assigned by the point-based scoring system, following the method described by Royston et al.^56^ We used severity levels to label the risk stages with stages 1 to 4, the higher the stages the greater likelihood of the occurrence of falls: stage 1 (PI ≤ 16th centile), stage 2 (PI 17-50th centile), stage 3 (PI 51-84th centile), and stage 4 (PI > 84th centile).

## Results

### Participants

The study sample included 5492 residents (70%, *n* = 3844 in the training and 30%, *n* = 1648 in the validation samples). Of the total sample, two-thirds (65.9%) were female, the median age was 86 years, and 53.4% had a dementia diagnosis at baseline. The median number of PH-FRAT assessments per resident over the follow-up was 5 (IQR 2-10). The demographic and health characteristics of validation sample were comparable to that of the training dataset in all variables. Medication usage was also comparable in both samples except for slightly higher utilization of antidementia drugs in the validation sample (Table 1).

**Table 1.** Participant characteristics in the training and validation cohort.

	All	Training	Validation	*P*
*N*	5492	3844	1648	
**Time-invariant variables**				
Female, *n* (%)	3617 (65.9)	2541 (66.1)	1076 (65.3)	
Age (years), median (IQR)	86.0 (81.0-91.0)	86.0 (81.0-91.0)	86.0 (81.0-91.0)	.955
Age group, *n* (%)				
65-80	1263 (23.0%)	892 (23.2%)	371 (22.5%)	.658
81-90	2811 (51.2%)	1952 (50.8%)	859 (52.1%)	
>90	1418 (25.8%)	1000 (26.0%)	418 (25.4%)	
Health status, *n* (%)				
Circulatory disease, any	4798 (87.4%)	3350 (87.1%)	1448 (87.9%)	.465
Hypertension	1998 (36.4%)	1378 (35.8%)	620 (37.6%)	.211
Cerebrovascular accident	1415 (25.8%)	981 (25.5%)	434 (26.3%)	.527
Endocrine, any	1937 (35.3%)	1365 (35.5%)	572 (34.7%)	.569
Diabetes	1320 (24.0%)	928 (24.1%)	392 (23.8%)	.778
Thyroid disorder	615 (11.2%)	436 (11.3%)	179 (10.9%)	.605
Chronic respiratory disease	970 (17.7%)	678 (17.6%)	292 (17.7%)	.943
Neoplasms/cancer	1486 (27.1%)	1037 (27.0%)	449 (27.2%)	.838
Dementia	2933 (53.4%)	2062 (53.6%)	871 (52.9%)	.591
Parkinson’s disease	438 (8.0%)	299 (7.8%)	139 (8.4%)	.411
Depression, mood, and affective disorders	2318 (42.2%)	1650 (42.9%)	668 (40.5%)	.100
Anxiety and stress-related disorders	1555 (28.3%)	1105 (28.7%)	450 (27.3%)	.278
PUD and GORD	1640 (29.9%)	1147 (29.8%)	493 (29.9%)	.955
Renal disease	964 (17.6%)	677 (17.6%)	287 (17.4%)	.861
Arthritis	3070 (55.9%)	2143 (55.7%)	927 (56.2%)	.732
Osteoporosis	1541 (28.1%)	1084 (28.2%)	457 (27.7%)	.723
Fracture	1900 (34.6%)	1331 (34.6%)	569 (34.5%)	.944
Hearing impairment	1346 (24.5%)	925 (24.1%)	421 (25.5%)	.242
Visual impairment	2455 (44.7%)	1727 (44.9%)	728 (44.2%)	.607
Falls history				
None in the last 12 months	1761 (32.1%)	1223 (31.8%)	538 (32.6%)	.068
≥1 in the last 3-12 months	1420 (25.9%)	1028 (26.7%)	392 (23.8%)	
≥1 in the last 3 months	947 (17.2%)	1593 (41.4)	718 (43.6)	
**Time-dependent variables^a^, *n* (%)**				
Severe psychological status^b^	998 (18.2%)	686 (17.8%)	312 (18.9%)	.339
Mobility/transfer issues	3965 (72.2%)	2776 (72.2%)	1189 (72.1%)	.959
Incontinence	3335 (60.7%)	2306 (60.0%)	1029 (62.4%)	.088
Risk-taking behaviors	3633 (66.2%)	2548 (66.3%)	1085 (65.8%)	.748
Environment	1603 (29.2%)	1112 (28.9%)	491 (29.8%)	.518
Nutrition	918 (16.7%)	666 (17.3%)	252 (15.3%)	.064
Medication (ever used/experienced over 5 years)				
Polypharmacy	4409 (80.3%)	3086 (80.3%)	1323 (80.3%)	.999
High sedative load	2631 (47.9%)	1831 (47.6%)	800 (48.5%)	.536
Opioids (N02A)	3432 (62.5%)	2397 (62.4%)	1035 (62.8%)	.754
Antipsychotics (N05A) excluding lithium	1640 (29.9%)	1162 (30.2%)	478 (29.0%)	.364
Antidepressants (N06A)	2558 (46.6%)	1818 (47.3%)	740 (44.9%)	.103
Anxiolytics (N05B)	609 (11.1%)	428 (11.1%)	181 (11.0%)	.870
Hypnotics and sedatives (N05C)	1825 (33.2%)	1285 (33.4%)	540 (32.8%)	.633
Antiepileptics (N03)	1405 (25.6%)	977 (25.4%)	428 (26.0%)	.666
Vasodilators (C01D)	552 (10.1%)	383 (10.0%)	169 (10.3%)	.742
Antihypertensives (C02)	204 (3.7%)	148 (3.9%)	56 (3.4%)	.417
Diuretics (C03)	2395 (43.6%)	1663 (43.3%)	732 (44.4%)	.429
Beta blockers (C07)	1752 (31.9%)	1230 (32.0%)	522 (31.7%)	.814
Calcium channel blockers (C08)	1218 (22.2%)	859 (22.3%)	359 (21.8%)	.646
Renin-angiotensin system inhibitor (C09)	2064 (37.6%)	1457 (37.9%)	607 (36.8%)	.453
Alpha adrenoceptor antagonist (G04CA)	271 (4.9%)	187 (4.9%)	84 (5.1%)	.716
Any analgesics (N02)	4905 (89.3%)	3429 (89.2%)	1476 (89.6%)	.693
Other analgesics and antipyretics (N02B)	4632 (84.3%)	3237 (84.2%)	1395 (84.6%)	.682
Anti-dementia drugs (N06D)	701 (12.8%)	466 (12.1%)	235 (14.3%)	.030
Anti-Parkinson drugs (N04)	483 (8.8%)	338 (8.8%)	145 (8.8%)	.995
Cardiac glycosides (C01A)	600 (10.9%)	415 (10.8%)	185 (11.2%)	.640
Antiarrhythmics, class I and III (C01B)	152 (2.8%)	102 (2.7%)	50 (3.0%)	.431
Lipid-modifying agents (C10)	2247 (40.9%)	1589 (41.3%)	658 (39.9%)	.330
Drugs for functional gastrointestinal disorders (A03)	1343 (24.5%)	913 (23.8%)	430 (26.1%)	.064
Proton pump inhibitors (A02BC)	2943 (53.6%)	2069 (53.8%)	874 (53.0%)	.591
Drugs for constipation (A06)	4052 (73.8%)	2830 (73.6%)	1222 (74.2%)	.683
Blood glucose-lowering drugs (A10B)	836 (15.2%)	582 (15.1%)	254 (15.4%)	.797
Insulins and analogues (A10A)	337 (6.1%)	235 (6.1%)	102 (6.2%)	.914
Antibacterials for systemic use (J01)	4554 (82.9%)	3171 (82.5%)	1383 (83.9%)	.198
Antineoplastic agents (L01)	226 (4.1%)	153 (4.0%)	73 (4.4%)	.442
Anti-inflammatory and antirheumatic products (M01)	713 (13.0%)	493 (12.8%)	220 (13.3%)	.596
Antigout preparations (M04)	439 (8.0%)	299 (7.8%)	140 (8.5%)	.369
Drugs for treatment of bone diseases (M05)	878 (16.0%)	610 (15.9%)	268 (16.3%)	.716
Drugs for obstructive airway diseases (R03)	1592 (29.0%)	1120 (29.1%)	472 (28.6%)	.711
Antihistamines for systemic use (R06)	685 (12.5%)	468 (12.2%)	217 (13.2%)	.308
Corticosteroids for systemic use (H02)	1083 (19.7%)	751 (19.5%)	332 (20.1%)	.603
Urologicals (G04)	333 (6.1%)	231 (6.0%)	102 (6.2%)	.798
Characteristics at the end of the study				
Total resident days	4 159 374	2 887 548	1 271 826	
Falls incidents				
Experienced a fall, *n* (%)	4271 (77.8)	2981 (77.6)	1290 (78.3)	.552
Experienced a recurrent fall, *n* (%)	3653 (66.5)	2561 (66.6)	1092 (66.3)	.795
Incident rate/1000 resident-day (95% CI)	7.76 (7.68-7.85)	7.72 (7.61-7.82)	7.88 (7.72-8.03)	.494
No. of falls per resident, median (IQR)	3 (1-7)	3 (1-7)	3 (1-8)	.453

a Ever experienced or ever used (in the case of medications). For example, the variable “Polypharmacy” signifies if a resident experienced polypharmacy at least once during the study period.

b Ever assessed as having severe form of one or more of anxiety, depression, cooperation, insight or judgement during the study period.

### Incidence of falls

**Figure 1.**
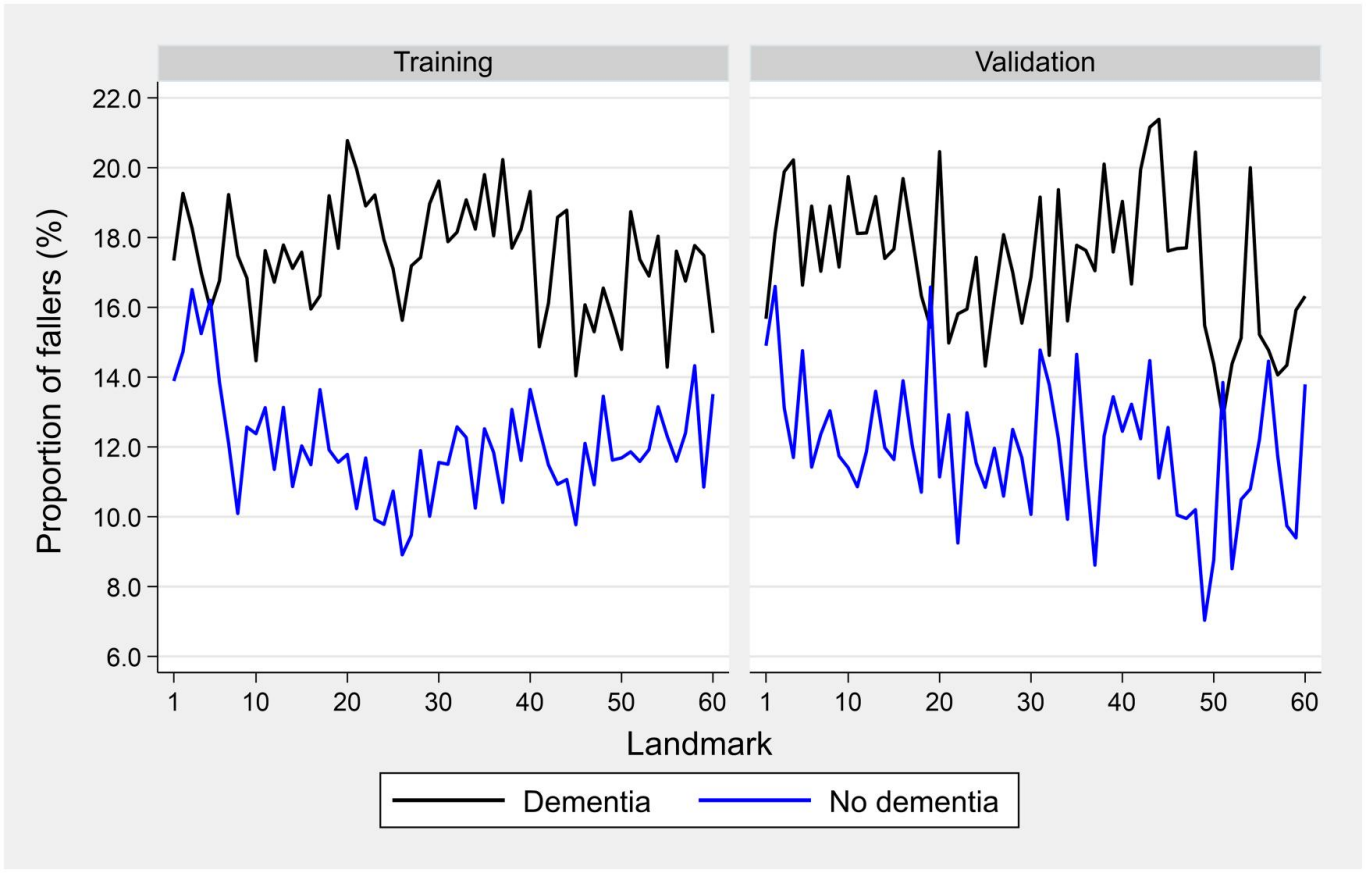
Proportion of residents with at least one fall by dementia status across landmarks. This longitudinal fall rate over five years suggests a comparable incidence of falls for both the training and validation samples in both cohorts.

A total of 32 296 falls were reported over 4 159 374 resident days resulting in a crude incident rate of 7.76 falls per 1000 resident days. Within the dementia cohort, the proportion of fallers varied across landmarks from 14.0% to 20.8% in the training sample and from 12.8% to 21.4% in the validation sample. For the nondementia cohort, fall rates ranged from 8.9% to 16.5% in the training sample and from 7.0% to 16.6% in the validation sample across the different landmarks. However, these proportions were consistently higher in the dementia versus nondementia cohorts in both the training and validation samples across all landmarks (Figure 1).

### Model development in the training sample

In the dementia cohort, 16 variables were significantly associated with an increased risk of falls, while 2 variables exhibited an inverse association. In the nondementia cohort, 13 variables exhibited a significant association with an increased risk of falls, while none showed an inverse association (Table S2). Additional details regarding various factors are described in the “Full model specification” section.

### Model performance in the validation sample

#### Dynamic AUROCC

**Figure 2.**
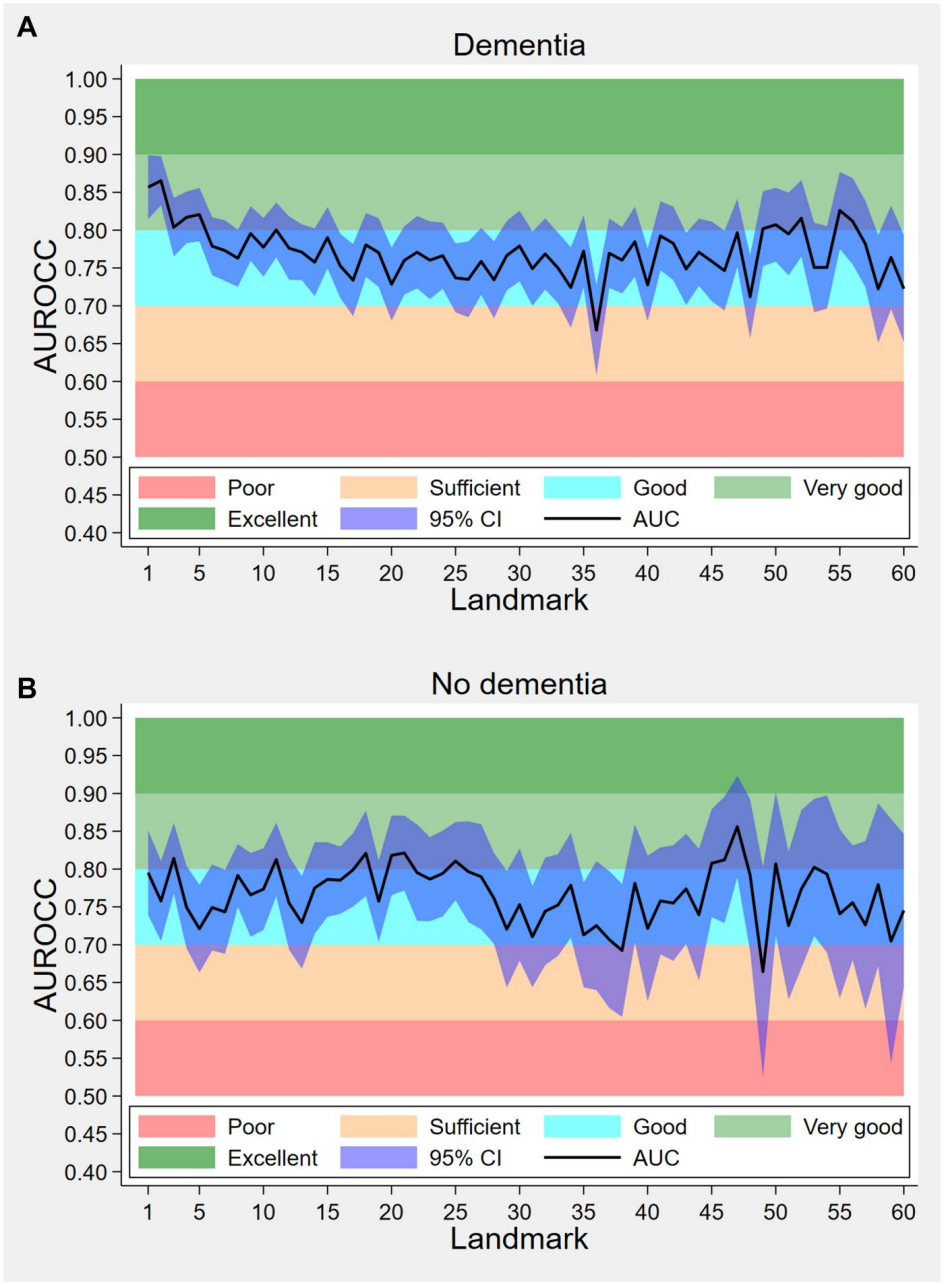
Dynamic AUROCC (landmark-specific) in residents (A) with and (B) without dementia in the validation sample. The plots of AUROCC per landmark were based on predicted probabilities; AUROCC: area under the curve.

**Figure 3.**
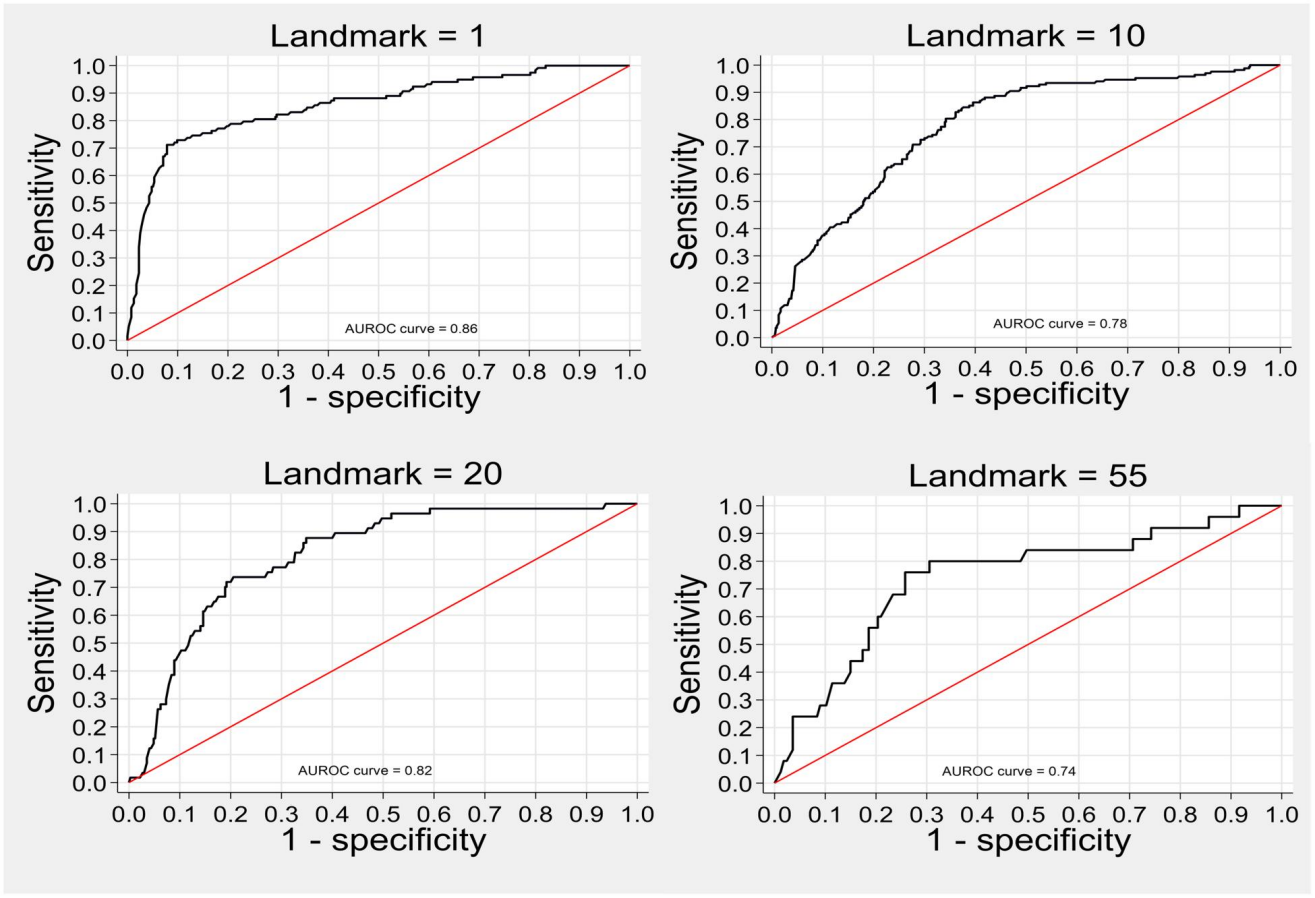
ROC curve for selected landmark in the validation sample. Landmarks 1 & 10: Dementia cohorts, Landmarks 20 & 55: Nondementia cohorts.

Figure 2 depicts the across landmarks. The AUROCC values exhibited fluctuations over time in both the dementia and nondementia cohorts; however, they primarily remained within the range of 0.75 to 0.85, which is considered indicative of “good” to “very good” discrimination. In the dementia cohort, 46 out of 60 landmarks had AUROCC values >0.75, with the highest at landmark 2, reaching 0.87. In the nondementia cohort, 40 out of 60 landmarks had AUROCC values >0.75, with the peak at landmark 47, reaching 0.86. Figure 3 shows the receiver operating characteristics (ROC) curve for selected landmarks. For instance, when examining landmark 1 from the dementia cohort, the ROC curve displayed an AUROCC of 0.86, indicating a “very good” discrimination (Figure 3A).

#### Dynamic sensitivity and specificity

**Figure 4.**
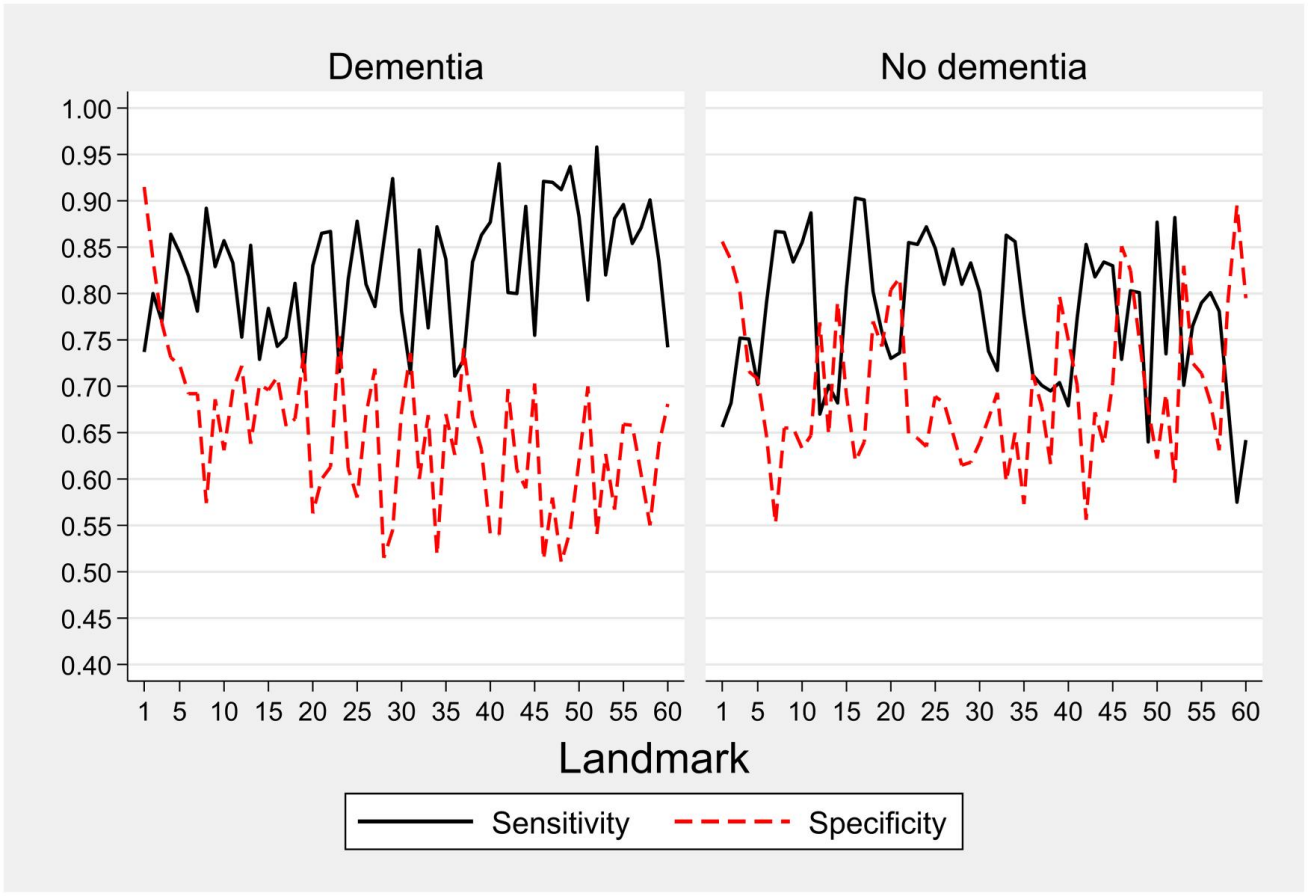
Sensitivity and specificity over time by dementia status in validation sample. The graph consistently shows high sensitivity and moderate specificity values for the models over time.

Figure 4 shows dynamic sensitivity and specificities. In the dementia cohort, sensitivity values ranged from 0.71 to 0.96, surpassing 0.80 in 42 out of 60 landmarks. In the nondementia cohort, sensitivity values ranged between 0.57 and 0.90, with over 0.8 observed in 30 out of 60 landmarks. These findings highlight the models’ robust sensitivity performance, underscoring their ability to accurately identify positive cases. On the other hand, the specificities were found to be moderate, with median values of 0.66 (range 0.51-0.92) for dementia and 0.68 (range 0.55-0.90) for nondementia cohorts.

### Full model specification

In the full model, we combined the training and validation samples and fit multivariate Cox landmark super models (Table 2). In the dementia cohort, 15 variables were significantly associated with an increased risk of falls, while one variable exhibited an inverse association. Interestingly, 3 variables (ie, osteoporosis/fracture, cardiac glycosides medication, and diuretics), which were initially found to be associated with falls in the training sample, did not maintain a significant association in the full sample. The variables linked to an increased risk of falls included being male, falls history, mobility/transfer issues, psychological problems, infection, and 8 medication-usage related variables. Falls history in the last 6 months was the strongest predictor of a subsequent fall [hazard ratio (HR) 4.75, 95% CI, 4.45-5.06, *P* < .001]. Of the medication classes, anti-Parkinson drug use had the highest impact: 51% higher risk in dementia (HR 1.37, 95% CI, 1.21-1.5) and 71% higher risk in nondementia cohorts (HR 1.71, 95% CI, 1.37-2.12). Renin-angiotensin system (RAS) inhibitors were the only cardiovascular drug found to be associated with an increased risk of falls (HR 1.33, 95% CI, 1.15-1.54). In contrast, the use of drugs for bone disease emerged as the only protective factor against falls, with its usage associated with a significant 21% lower risk of falls compared to nonusers (HR 0.79, 95% CI, 0.66-0.96).

**Table 2.** Multivariate cox landmark supermodel in full sample.

	Dementia				No dementia			
	Coefficient (β)	HR (95% CI)	*P*	Points	Coefficient (β)	HR (95% CI)	*P*	Points
Male vs female	0.26	1.29 (1.19-1.41)	<.001	3	0.32	1.38 (1.25-1.53)	<.001	3
**Age group (Ref = 65-80 years)**								
81-90	−0.01	0.99 (0.89-1.11)	.910	0	0.29	1.34 (1.11-1.61)	<.001	3
>90	0.07	1.07 (0.93-1.22)	.350	0	0.28	1.33 (1.12-1.57)	<.001	3
Cerebrovascular accident	0.04	1.04 (0.96-1.14)	.330	0	0.05	1.05(0.94-1.18)	.410	0
Visual impairment	0.04	1.04 (0.94-1.15	.480	0	−0.02	0.98 (0.88-1.10)	.780	0
**Falls history at admission (Ref = none in the last 12 months)**								
≥1 in the last 3-12 months	0.16	1.18 (1.06-1.3)	<.001	2	0.19	1.21 (1.07-1.36)	<.001	2
≥1 in the last 3 months	0.35	1.42 (1.30-1.56)	<.001	4	0.45	1.56 (1.39-1.75)	<.001	5
Mobility/transfer issues	0.27	1.31 (1.22-1.40)	<.001	3	0.27	1.31 (1.19-1.44)	<.001	3
Osteoporosis/fracture	0.06	1.06 (0.99-1.15)	.120	0	0.07	1.08 (0.97-1.19)	.160	0
Incontinence	−0.06	0.94 (0.87-1.01)	.110	0	−0.07	0.93 (0.84-1.03)	.160	0
**Psychological status^a^ (Ref = no)**								
Mild	0.11	1.11 (0.98-1.26)	.100	0	0.2	1.22 (1.10-1.36)	<.001	2
Moderate	0.25	1.29 (1.13-1.47)	<.001	3	0.46	1.59 (1.37-1.84)	<.001	5
Severe	0.48	1.62 (1.40-1.88)	<.001	5	0.76	2.14 (1.77-2.58)	<.001	8
Fell in the last 6 months prior to an episode of falls in a landmark^b^	1.56	4.75 (4.45-5.06)	<.001	16	1.44	4.20 (3.87-4.57)	<.001	14
Infection^c^	0.38	1.47 (1.36-1.59)	<.001	4	0.27	1.31(1.20-1.42)	<.001	3
**No. of medications (Ref = none)**								
1-4	0	1.00 (0.86-1.17)	.990	0	0.08	1.09 (0.92-1.28)	.340	0
5-8	−0.03	0.97 (0.84-1.13)	.710	0	−0.06	0.94 (0.79-1.11)	.480	0
≥9	−0.13	0.88 (0.74-1.03)	.110	0	−0.16	0.85 (0.69-1.05)	.130	0
Anti-Parkinson drugs (N04)	0.41	1.51 (1.27-1.78)	<.001	4	0.53	1.71(1.37-2.12)	<.001	5
Drugs for bone diseases (M05)	−0.23	0.79 (0.66-0.96)	.010	−2	−0.09	0.91(0.72-1.15)	.440	0
Corticosteroids for systemic use (H02)	0.15	1.16 (1.00-1.34)	.050	2	−0.04	0.96 (0.82-1.13)	.620	0
Drugs for constipation (A06)	−0.01	0.99 (0.92-1.06)	.720	0	−0.03	0.97 (0.87-1.08)	.540	0
Cardiac glycosides (C01A)	0.14	1.16 (0.99-1.35)	.070	0	0.11	1.12 (0.92-1.35)	.260	0
Urologicals (G04)	0.01	1.01 (0.89-1.15)	.850	0	0.01	1.01 (0.87-1.18)	.850	0
Lipid modifying agents (C10)	−0.06	0.94 (0.86-1.03)	.200	0	−0.03	0.97 (0.87-1.08)	.530	0
**Analgesics (Ref = no)**								
Other analgesics and antipyretics (N02B)	0.2	1.22 (1.13-1.32)	<.001	2	0.11	1.12 (1.00-1.25)	.050	1
Opioids (N02A)	0.24	1.27 (1.16-1.39)	<.001	2	0.14	1.15 (1.02-1.30)	.020	1
**Anxiolytics/hypnotics/sedatives (Ref = no)**								
Existing users since the last LM	0.12	1.13 (1.02-1.26)	.020	1	0.06	1.07 (0.96-1.18)	.240	0
New users in the current LM	0.35	1.41 (1.24-1.61)	<.001	4	0.18	1.19 (1.02-1.39)	.030	2
**Antidepressants (Ref = no)**								
Existing users since the last LM	0.09	1.09 (1.01-1.18)	.030	1	0.01	1.01 (0.90-1.14)	.850	0
New users in the current LM	0.31	1.37 (1.21-1.54)	<.001	3	0.2	1.22 (1.03-1.46)	.020	2
**Antipsychotics (Ref = no)**								
Existing users since the last LM	0.12	1.12 (1.03-1.22)	.010	1	0.07	1.07 (0.91-1.27)	.410	0
New users in the current LM	0.22	1.24 (1.07-1.44)	<.001	2	0.33	1.39 (1.10-1.75)	.010	3
**Antiepileptics (Ref = no)**								
Existing users since the last LM	0.16	1.17 (1.04-1.32)	.010	2	0.04	1.04 (0.90-1.21)	.590	0
New users in the current LM	0.21	1.24 (1.04-1.47)	.010	2	0.14	1.15 (0.93-1.42)	.200	0
**Diuretics (Ref = no)**								
Existing users since the last LM	−0.07	0.93 (0.86-1.01)	.090	0	−0.1	0.91 (0.82-1.01)	.080	0
New users in the current LM	0.01	1.01 (0.87-1.16)	.940	0	0.13	1.14 (0.97-1.34)	.120	0
**RAS inhibitor (Ref = no)**								
Existing users since the last LM	−0.07	0.93 (0.86-1.02)	.120	0	−0.07	0.94 (0.82-1.07)	.340	0
New users in the current LM	0.28	1.33 (1.15-1.54)	<.001	3	0.03	1.03 (0.79-1.35)	.810	0

a Severity of psychological assessment in one or more of anxiety, depression, cooperation, insight or judgement during the study period.

b One or more falls in the last 6 months after admission into RACFs but prior to an episode of falls in each landmark.

c The use of systemic antibiotics was used as a proxy measure of infection. RAS, renin-angiotensin system.

In the nondementia cohort, 12 variables exhibited a significant association with an increased risk of falls, while none showed an inverse association. Drugs used for bone diseases, corticosteroids, antiepileptics, and RAS inhibitors, which were significant in the dementia cohort, did not demonstrate a significant association with falls in the nondementia cohort. In contrast to the dementia cohort, however, age was significantly associated with an increased risk of falls in the nondementia cohort.

### Development of a point-based risk-scoring system and fall risk staging

To complete the monitoring component of the FRIPAC tool, a point-scoring system was developed based on the coefficients of each significant variable in the full model (Table 2).^54^^,^^55^ The theoretical range of total points were from −2 to 57 in dementia and 0 to 52 in nondementia cohorts. A score of “−2” in the dementia cohort signifies the protective effects linked to the medication administered for bone disease. In both cohorts, falls history since admission (ie, *fell in the last 6 months prior to an episode of falls in a landmark*) received the highest point allocation, with 16 points in the dementia cohort and 14 points in the nondementia cohort. Notably, medication-related variables collectively accounted for a significant portion of the point system, representing nearly half of the total possible points (28 points, 47.5%) in the dementia cohort. In contrast, in the nondementia cohort, medication-related factors accounted for 30.8% of the total possible points (16 out of 52). These findings underscore the substantial influence of falls history and medication-related factors in assessing and predicting fall risk in both dementia and nondementia populations. An example of how to estimate the risk of falls is provided in Box S3.

**Figure 5.**
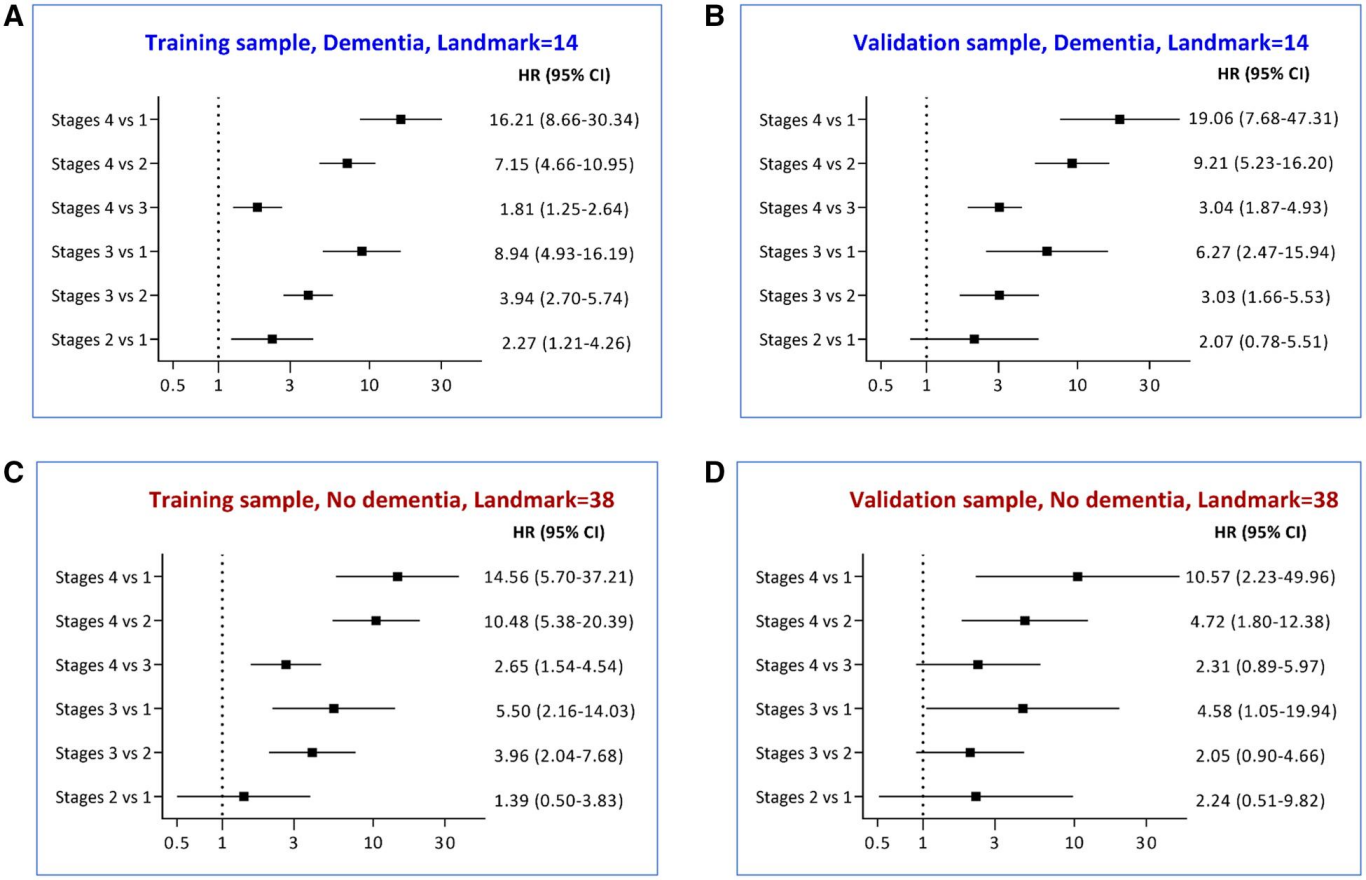
Pairwise comparisons of fall risk groups in the training and validation sample using landmarks 14 and 38 as examples. Note: The results above indicate the hazard ratio of pair-wised comparisons of risk stages for selected landmarks in the training (A and C) and validation (B and D) sample. For instance, in (B), residents at risk stage 4 had ∼19 times higher likelihood of experiencing a fall compared to those at risk stage 1 in validation sample.

A risk staging system consisting of 4 stages (1-4) was established by utilizing the 16th, 50th, and 84th centiles of the PI. These risk stages are not fixed and can change monthly, depending on an individual’s risk status. This dynamic nature allows for individuals to potentially move across different risk stages over time. Figure 5A-D shows pairwise comparisons of risk stages in the training and validation sample for selected landmarks. For instance, in Figure 5B, residents at risk stage 4 had ∼19 times higher likelihood of experiencing a fall compared to those at-risk stage 1.

## Discussion

We developed a novel dynamic fall risk prediction and monitoring tool that harnesses the wealth of information within routinely collected electronic health records. Our final models emerged from rigorous testing of over 100 variables, identifying 15 and 12 independent predictors of falls in dementia and nondementia cohorts, respectively. Using a point-based risk-scoring system, we transformed the results into 4 risk stages to enable continuous monitoring of fall risk in a highly interpretable manner. This supports the proactive design and implementation of fall prevention and intervention strategies, aimed at reducing fall risk among vulnerable individuals.

Our findings highlight the superior performance of FRIPAC, compared to currently used tools as AUROCC values mostly occur within the 0.75-0.85 range and sensitivity mostly surpass 80%. This is likely clinically significant as current tools have poor predictive power. Notably, the widely used PH-FRAT in RACFs exhibits a poor AUROCC value of 0.57.^57^ In past research, the sensitivity of the PH-FRAT (52%-58%) and other commonly used fall assessment tools in Australian RACFs, such as the modified fall assessment tool (61%), were consistently lower than that achieved by FRIPAC.^40^ The landmark approach enables the efficient utilization of time-dependent, repeated measurements of crucial fall risk factors that naturally evolve over time,^58^ leading to the improved performance of FRIPAC. Although some studies reported high sensitivities of certain fall risk assessment tools, concerns arise due to their small sample sizes and suboptimal methodologies.^40^

Our study integrates 2 distinct tools to predict falls, acknowledging dementia status as a significant risk factor.^59^ Individuals with dementia share many fall risk factors as those without, but their poorer performance on these factors warrants separate tools tailored to their specific risk profiles. This is evidenced by a notably higher proportion of dementia residents experiencing at least one fall across all landmarks compared to nondementia patients within the study sample. It is observed that medications for bone diseases, systemic corticosteroids, antiepileptics, and RAS inhibitors significantly heighten fall risk in individuals with dementia but not in those without, underscoring the importance of considering dementia status in assessing their impact. In contrast, age emerged as a risk factor for falls in the nondementia cohort but not in the dementia cohort. This suggests the presence of dementia negates the influence of age as a contributing factor to fall risk.

In the present study, aligning with international literature across diverse settings, falls history emerged as the strongest predictor of future falls. Our study, however, underscores the significance of considering falls history as a dynamic time-dependent risk factor, focusing on falls occurring within the last 6-month period. This challenges the notion that once residents experience a fall, they are at immediate risk of another. Our approach recognizes residents’ potential for improvement, focusing on recent falls history, and acknowledging that a fall several months ago may not necessarily affect current care. Findings of other independent predictor variables in the FRIPAC, such as mobility issues, psychotropic medications, anti-Parkinson drugs, and poor psychological status, are consistent with international literature.^16^^,^^60^^,^^61^

Our findings challenge the assumption that links to the use of antihypertensive medications to falls. Except for individuals with dementia who had recently initiated RAS inhibitors, the use of other antihypertensive medications, did not demonstrate associations with falls. This is noteworthy, considering their classification as FRIDs^62^ and their incorporation into certain existing fall risk assessment tools.^63–65^ The presumed mechanism causing falls involves the development of orthostatic hypotension—marked drops in blood pressure when upright—which may lead to insufficient blood flow to the brain, resulting in fainting.^66^ Some pharmaco-epidemiological studies, in line with our findings, have also reported the absence of associations between antihypertensive medications and falls in older people.^67^^,^^68^ In fact, some studies have even indicated a protective effect for some FRIDs (eg, beta-blockers).^69^ While we acknowledge that antihypertensive medications can induce postural hypotension, their impact on falls might be minimal when other factors (eg, history of falls) are taken into consideration in the RACF population, since individuals in RACFs are less mobile, their exposure to the orthostatic effect of medication may be limited.

We adopted staging terminology to categorize fall risk groups as stages 1-4, deliberately avoiding the conventional low-medium-high terminology used in previous tools. This adaptation acknowledges that the prevalence of falls is very high in RACFs^45^ and that all RACF residents inherently carry a “high risk,”^61^ yet the quantifiable fall risk may vary which is better represented through distinct stages. Implementing this staging strategy not only streamlines risk assessment but also identifies personalized risk profiles and modifiable risk factors. This approach reduces the labor intensity associated with conducting multifactorial assessments, as dynamic identification of modifiable individual risk factors (eg, FRIDs) is accomplished through routinely collected data. Furthermore, the point-based scoring system, in accordance with the model coefficients, enables caregivers to monitor residents and identify the individualized risk profiles by utilizing data values for the modeled variables, even in the absence of routinely collected data.

### Implications for policy, practice, and future research

The FRIPAC tool has strong implications for policies and practices. The FRIPAC is highly applicable and has the potential to be used in various medical and healthcare settings where basic electronic individual data are available. We transformed the complex landmark model into an easy-to-interpret point-based risk scoring system. This aims to facilitate seamless implementation in healthcare settings equipped with electronic health records, ensuring the practical relevance and applicability of FRIPAC in real-world settings. The point-based scoring system also enables external validation of the tool, providing researchers with the necessary information to benchmark FRIPAC against other fall prediction tools. Practically, the FRIPAC may be useful to identify a change in fall risk from high to higher and in a resource-poor setting to stratify risk so that staff may prioritize fall prevention interventions and assistance. The automated functionality of the FRIPAC offers the advantage of reducing the documentation workload on staff members, who would otherwise spend a substantial time completing a fall risk assessment.^45^ Given the shortage of trained aged care health professionals in Australia and in many other industrialized countries, the automated nature of the FRIPAC may reduce the documentation burden on staff who traditionally need to manually complete a fall risk assessment. However, it is crucial to note that while automation can aid in efficiency, it should not replace the clinical judgment of trained healthcare professionals in aged care.

The FRIPAC was developed as a part of a large grant from National Health and Medical Research Council (NHMRC). This grant aims to implement a falls prevention dashboard, which will support the 3 pillars of falls prevention through incorporating the predictive model (ie, pillar two: risk assessment of individual-level risk factors through routinely collected data to identify individuals’ likelihood of experiencing a fall) and visualizing various individual- and facility-level risk factors associated with falls (ie, for supporting pillar two: risk assessment of individual and physical environment and pillar one: awareness for care givers on risk factors). Most importantly, the dashboard will also provide falls prevention intervention recommendations to guide aged care staff for effective falls prevention (ie, supporting pillar 3: promoting multifactorial interventions). These recommendations are personalized for individuals’ risk profile by integrating the fall risk identified through the predictive tool and other falls-related risk factors (eg, the most common locations and times of falls and changes observed in the administration of drugs that increase fall risk) obtained from visualization.

### Study limitations and future direction

The current study has several strengths, including its large sample size, multiple facilities, dynamic nature, and the use of readily available electronic data. Notably, the incorporation of routinely collected electronic data eliminates the need for manual data collection. However, our study has some limitations. Firstly, while both intrinsic and extrinsic factors are associated with falls,^16^ our study exclusively utilizes intrinsic variables extracted from electronic health records. Data limitations restricted the inclusion of relevant extrinsic variables such as environment factors (eg, flooring quality). Secondly, the comorbidities utilized in the study were based on data recorded at admission, which may overlook potentially relevant conditions that could develop after admission. Our 2 models were based on dementia diagnoses recorded at baseline. This could introduce misclassification bias if residents in nondementia cohorts develop dementia postadmission. Thirdly, the 2 variables in the final model from the PH-FRATs—namely, psychological status and mobility status—might not be updated before a falls event if there has been no recent assessment. Therefore, the study encourages aged care facilities and software providers to collect these variables at admission and update them routinely as they change. Fourthly, while we have performed internal validation of the FRIPAC obtaining satisfying results, this tool needs to be externally validated and recalibrated for further evidence regarding its validity, accuracy, and reliability. Furthermore, since some risk factors identified in our study are nonmodifiable, it is important to determine the effectiveness of interventions based on our models in reducing actual falls.

## Conclusions

The study introduces the FRIPAC, a dynamic fall risk prediction and monitoring tool developed through an innovative landmarking method that leverages the wealth of information within routinely collected electronic health records. We developed separate tools based on dementia status, recognizing dementia as a critical risk factor in RACFs. FRIPAC generates personalized, dynamic fall risk predictions, allowing for continuous and easily interpretable monitoring of fall risk in RACFs and the implementation of targeted interventions. Embedding these tools within electronic health records would significantly enhance the capacity of healthcare professionals and care providers for falls management, aligning with the growing emphasis on care quality and digitalization in RACFs.

## Supplementary Material

ocae058_Supplementary_Data

## Data Availability

The data underlying this article cannot be shared publicly due to for the privacy of individuals that participated in the study. The data will be shared on reasonable request to the corresponding author. Researchers interested in implementing FRIPAC and in need of additional information, including details on landmark-specific baseline survival data, can contact the authors for further details.
