# Supplementary material for: Development and internal validation of a dynamic fall risk prediction and monitoring tool in aged care using routinely collected electronic health data: a landmarking approach
Source: J Am Med Inform Assoc. 2024 Mar 26;31(5):1113–25. doi: 10.1093/jamia/ocae058 (PMC11031240; doi:10.1093/jamia/ocae058)
Supplement: ocae058_Supplementary_Data [file ocae058_supplementary_data.docx]

# Supplementary File

**Supplementary Table 1: Candidate predictors considered in the model.**

| *Data source and variables* | Variable  type | Time  dependency | Definition |
| --- | --- | --- | --- |
| *Resident profile – demographics* |  |  |  |
| Sex | Binary (M, F) | Time-invariant |  |
| Age | Continuous | Time-invariant |  |
| *Resident profile – health status* |  |  |  |
| Circulatory disease, any | Binary (Y, N) | Time-invariant |  |
| Hypertension | Binary (Y, N) | Time-invariant |  |
| Cerebrovascular accident | Binary (Y, N) | Time-invariant |  |
| Endocrine, any | Binary (Y, N) | Time-invariant |  |
| Diabetes | Binary (Y, N) | Time-invariant |  |
| Thyroid disorder | Binary (Y, N) | Time-invariant |  |
| Chronic respiratory disease | Binary (Y, N) | Time-invariant |  |
| Neoplasms | Binary (Y, N) | Time-invariant |  |
| Dementia | Binary (Y, N) | Time-invariant |  |
| Parkinson’s disease | Binary (Y, N) | Time-invariant |  |
| Depression, mood & affective disorders | Binary (Y, N) | Time-invariant |  |
| Anxiety & stress-related disorders | Binary (Y, N) | Time-invariant |  |
| PUD & GORD | Binary (Y, N) | Time-invariant |  |
| Renal disease | Binary (Y, N) | Time-invariant |  |
| Arthritis | Binary (Y, N) | Time-invariant |  |
| Osteoporosis | Binary (Y, N) | Time-invariant |  |
| Fracture | Binary (Y, N) | Time-invariant |  |
| Hearing impairment | Binary (Y, N) | Time-invariant |  |
| Visual impairment | Binary (Y, N) | Time-invariant |  |
| Falls history | Categorical | Time-invariant | Three categories: None in the last 12 months; ≥1 in the last 3-12 months and ≥1 in the last 3 months |
| *Medication administration* |  |  |  |
| Polypharmacy | Binary (Y, N) | Time-varying | >9 regular medications |
| Sedative load | Continuous | Time-varying | A metrics to quantify the combined effect of exposure to medications with sedative properties |
| Opioids | Binary (Y, N) | Time-varying | ATC code N02A |
| Antipsychotics | Binary (Y, N) | Time-varying | ATC code N05A excluding lithium |
| Antidepressants | Binary (Y, N) | Time-varying | ATC code N06A |
| Anxiolytics | Binary (Y, N) | Time-varying | ATC code N05B |
| Hypnotics and sedatives | Binary (Y, N) | Time-varying | ATC code N05C |
| Antiepileptics | Binary (Y, N) | Time-varying | ATC code N03 |
| Vasodilators | Binary (Y, N) | Time-varying | ATC code C01D |
| Antihypertensives | Binary (Y, N) | Time-varying | ATC code C02 |
| Diuretics | Binary (Y, N) | Time-varying | ATC code C03 |
| Beta blockers | Binary (Y, N) | Time-varying | ATC code C07 |
| Calcium channel blockers | Binary (Y, N) | Time-varying | ATC code C08 |
| Renin-angiotensin system inhibitor | Binary (Y, N) | Time-varying | ATC code C09 |
| Alpha adrenoceptor antagonist | Binary (Y, N) | Time-varying | ATC code G04CA |
| Any analgesics | Binary (Y, N) | Time-varying | ATC code N02 |
| Other analgesics & antipyretics | Binary (Y, N) | Time-varying | ATC code N02B |
| Anti-dementia drugs | Binary (Y, N) | Time-varying | ATC code N06D |
| Anti-Parkinson drugs | Binary (Y, N) | Time-varying | ATC code N04 |
| Cardiac glycosides | Binary (Y, N) | Time-varying | ATC code C01A |
| Antiarrhythmics, class I & III | Binary (Y, N) | Time-varying | ATC code C01B |
| Lipid modifying agents | Binary (Y, N) | Time-varying | ATC code C10 |
| Drugs for functional gastrointestinal disorders | Binary (Y, N) | Time-varying | ATC code A03 |
| Proton Pump Inhibitors | Binary (Y, N) | Time-varying | ATC code A02BC |
| Drugs for constipation | Binary (Y, N) | Time-varying | ATC code A06 |
| Blood glucose lowering drugs | Binary (Y, N) | Time-varying | ATC code A10B |
| Insulins & analogues | Binary (Y, N) | Time-varying | ATC code A10A |
| Antibacterials for systemic use | Binary (Y, N) | Time-varying | ATC code J01 |
| Antineoplastic agent | Binary (Y, N) | Time-varying | ATC code L01 |
| Anti-inflammatory & antirheumatic products | Binary (Y, N) | Time-varying | ATC code M01 |
| Antigout preparations | Binary (Y, N) | Time-varying | ATC code M04 |
| Drugs for treatment of bone diseases | Binary (Y, N) | Time-varying | ATC code M05 |
| Drugs for obstructive airway diseases | Binary (Y, N) | Time-varying | ATC code R03 |
| Antihistamines for systemic use | Binary (Y, N) | Time-varying | ATC code R06 |
| Corticosteroids for systemic use | Binary (Y, N) | Time-varying | ATC code H02 |
| Urologicals | Binary (Y, N) | Time-varying | ATC code G04 |
| PH-FRAT database |  |  |  |
| Psychological status | Categorical  (4 groups*) | Time-varying | Having one or more of anxiety, depression, cooperation, insight or judgement |
| Functional status | Binary (Y, N) | Time-varying | Change in functional status including dizziness & postural hypotension since the last assessment |
| Mobility/transfer issues | Binary (Y, N) | Time-varying | E.g., the use of a 4-wheel walker |
| Risk-taking behaviours | Binary (Y, N) | Time-varying | Observed/reported unsafe use of equipment, unsafe footwear |
| Environment | Binary (Y, N) | Time-varying | Observed/reported difficulties with orientation to the environment |
| Nutrition | Binary (Y, N) | Time-varying | Underweight/low appetite |

*Minor, mild, moderate and severe

**Supplementary Box 1: Creating a landmark super prediction dataset.**

| We applied the following four steps, in which all residents were followed from admission for 60 months in RACFs to accommodate at least 95th percentile of the length of stays of permanent residents of RACFs. (1) Firstly, we set a 1-monthly uniformly spaced time points (*hereafter* ‘landmark’ times) resulting in up to 60 landmark months. (2) Then, from each landmark, we adopted a prediction window or horizon of 1 month, yielding a non-overlapping landmark times over the 5-year follow-up period. (3) After that for each landmark, a separate dataset was created by left truncation and administrative censoring at the end of each prediction window. This results in up to 60 data rows for a given resident. A given landmark contains residents who were active and at risk (that is, residents who were still in the facilities at the beginning of a new landmark) regardless of whether they experienced a fall in the previous landmark or not. Information of time-dependent variables were updated at each landmark. We used a look back period of three days to define medication use status at each landmark. For instance, antipsychotic medication use status at landmark 3 was defined as whether a resident received antipsychotics in the last three days prior to the start of landmark 3. We used the *last observation carried forward* approach for other time-dependant variables. (4) Finally, all landmark-specific datasets created in step 3 were stacked to create a large super prediction dataset. This dataset was then used to fit a model. We applied a *stratified landmark supermodel*, a type of extended Cox model that allows separate baseline hazards for each landmark and common predictor coefficients across all landmarks, to dynamically estimate the probability of experiencing a fall in each landmark. We used robust standard errors, as the same residents can appear multiple times in the super prediction dataset, when fitting the model. |
| --- |

**Supplementary Box 2: Model development and internal validation**

| The training sample was used for model development and the validation sample was used for internal validation (i.e., to measure model performance). The splitting of the super prediction dataset was stratified by landmark to ensure that data from the same landmark for a given resident appear either in the training or validation sample but not in both samples. The characteristics of training versus validation samples were compared using the χ2 statistics or Wilcoxon rank-sum test.  Our variable selection methodology was adopted from Collett's^1^ approach and followed the following steps. Firstly, we fitted a univariate stratified landmark supermodel and predictors significant at P = 0.20 were identified for the next step. Secondly, all significant predictors from step 1 were used to fit a multivariate stratified landmark supermodel. At this step, we used backward selection to eliminate non-significant variables at P = 0.10. Thirdly, beginning with the final model from step 2, each of the non-significant variables from step 1 were considered and forward selection was used to retain predictors significant at P = 0.10. The final step involved pruning of main and interactions effects using stepwise regression at P = 0.05. Whilst we identified significant interaction effects between some variables in our model, the gain in the model performance was minute. Thus, we opted not to include interaction effects in our final model for the sake of parsimony. The final model was used to generate predicted probabilities in the validation sample.  **References**  1. Collett D. Modelling Survival Data in Medical Research. 1993 |
| --- |

**Supplementary Table 2: Multivariate cox landmark supermodel in the training sample.**

|  | Dementia | | No dementia | |
| --- | --- | --- | --- | --- |
|  | HR (95% CI) | P | HR (95% CI) | P |
| Male vs Female | 1.34 (1.22-1.47) | 0.000 | 1.31 (1.17-1.47) | 0.000 |
| Age group (Ref=65-80 years) |  |  |  |  |
| 81-90 | 1.06 (0.94-1.21) | 0.330 | 1.51 (1.28-1.77) | 0.000 |
| >90 | 1.09 (0.93-1.27) | 0.270 | 1.54 (1.32-1.80) | 0.000 |
| Cerebrovascular accident | 1.07 (0.96-1.18) | 0.230 | 1.09 (0.95-1.25) | 0.210 |
| Visual impairment | 1.09 (0.96-1.24) | 0.180 | 1.00 (0.89-1.13) | 0.950 |
| Falls history at admission |  |  |  |  |
| ≥1 in the last 3-12 months | 1.21 (1.07-1.37) | 0.000 | 1.22 (1.05-1.41) | 0.010 |
| ≥1 in the last 3 months | 1.39 (1.25-1.55) | 0.000 | 1.56 (1.36-1.78) | 0.000 |
| Mobility/transfer issues | 1.30 (1.20-1.42) | 0.000 | 1.28 (1.15-1.43) | 0.000 |
| Osteoporosis/fracture | 1.10 (1.00-1.20) | 0.050 | 1.04 (0.92-1.18) | 0.500 |
| Incontinence | 0.95 (0.86-1.04) | 0.230 | 0.93 (0.82-1.06) | 0.290 |
| Psychological status^1^ (Ref=No) |  |  |  |  |
| Mild | 1.11 (0.96-1.29) | 0.170 | 1.17 (1.03-1.33) | 0.020 |
| Moderate | 1.28 (1.09-1.50) | 0.000 | 1.62 (1.37-1.91) | 0.000 |
| Severe | 1.60 (1.34-1.91) | 0.000 | 2.10 (1.66-2.66) | 0.000 |
| Fell in the last 6 months prior to a LM^2^ | 4.58 (4.27-4.92) | 0.000 | 4.13 (3.75-4.54) | 0.000 |
| Infection^3^ | 1.54 (1.40-1.69) | 0.000 | 1.40 (1.27-1.55) | 0.000 |
| No. of medications (Ref=None) |  |  |  |  |
| 1-4 | 1.05 (0.89-1.22) | 0.570 | 1.06 (0.87-1.29) | 0.550 |
| 5-8 | 1.06 (0.90-1.24) | 0.500 | 0.89 (0.74-1.06) | 0.190 |
| ≥9 | 0.90 (0.75-1.08) | 0.260 | 0.77 (0.63-0.95) | 0.010 |
| Anti-Parkinson drugs (N04) | 1.27 (1.06-1.52) | 0.010 | 1.59 (1.28-1.97) | 0.000 |
| Drugs for bone diseases (M05) | 0.79 (0.63-1.00) | 0.050 | 0.98 (0.72-1.33) | 0.910 |
| Corticosteroids for systemic use (H02) | 1.24 (1.05-1.46) | 0.010 | 0.98 (0.81-1.19) | 0.830 |
| Drugs for constipation (A06) | 0.97 (0.89-1.05) | 0.420 | 0.97 (0.87-1.07) | 0.520 |
| Cardiac glycosides (C01A) | 1.22 (1.01-1.47) | 0.040 | 1.14 (0.90-1.46) | 0.280 |
| Urologicals (G04) | 1.07 (0.92-1.25) | 0.370 | 1.01 (0.85-1.20) | 0.920 |
| Lipid modifying agents (C10) | 0.96 (0.87-1.07) | 0.480 | 0.97 (0.85-1.11) | 0.650 |
| Analgesics (Ref=None) |  |  |  |  |
| Other analgesics & antipyretics (N02B) | 1.20 (1.09-1.31) | 0.000 | 1.15 (1.01-1.30) | 0.030 |
| Opioids (N02A) | 1.32 (1.19-1.47) | 0.000 | 1.27 (1.10-1.47) | 0.000 |
| Anxiolytics/Hypnotics/Sedatives (Ref=No) |  |  |  |  |
| Existing users since the last LM | 1.15 (1.01-1.30) | 0.030 | 1.03 (0.91-1.16) | 0.670 |
| New users in the current LM | 1.35 (1.16-1.58) | 0.000 | 1.15 (0.96-1.38) | 0.140 |
| Antidepressants (Ref=No) |  |  |  |  |
| Existing users since the last LM | 1.11 (1.01-1.22) | 0.030 | 1.00 (0.88-1.13) | 0.980 |
| New users in the current LM | 1.43 (1.24-1.64) | 0.000 | 1.23 (1.00-1.51) | 0.050 |
| Antipsychotics (Ref=No) |  |  |  |  |
| Existing users since the last LM | 1.23 (1.12-1.36) | 0.000 | 1.21 (1.01-1.45) | 0.040 |
| New users in the current LM | 1.23 (1.04-1.45) | 0.010 | 1.51 (1.15-1.98) | 0.000 |
| Antiepileptics (Ref=No) |  |  |  |  |
| Existing users since the last LM | 1.19 (1.04-1.36) | 0.010 | 1.14 (0.95-1.38) | 0.160 |
| New users in the current LM | 1.26 (1.04-1.53) | 0.020 | 1.19 (0.91-1.55) | 0.200 |
| Diuretics (Ref=No) |  |  |  |  |
| Existing users since the last LM | 0.91 (0.82-0.99) | 0.040 | 0.86 (0.77-0.97) | 0.010 |
| New users in the current LM | 1.07 (0.91-1.27) | 0.410 | 1.18 (0.97-1.43) | 0.100 |
| RAS inhibitor (Ref=No) |  |  |  |  |
| Existing users since the last LM | 0.92 (0.83-1.02) | 0.100 | 0.88 (0.76-1.02) | 0.090 |
| New users in the current LM | 1.30 (1.10-1.55) | 0.000 | 0.89 (0.70-1.12) | 0.320 |

^1^Severity of psychological assessment in one or more of anxiety, depression, cooperation, insight, or judgement during the study period. ^2^One or more falls in the last six months after admission into RACFs but prior to an episode of falls in each landmark. ^3^The use of systemic antibiotics use was used as a proxy measure of infection. RAS, Renin-angiotensin system.

**Supplementary Box 3: Example of how to estimate risk of falls using the landmarking approach**

| The estimated risk associated with total points can be calculated using the formula$\boldsymbol{1-s}\boldsymbol{0}\left( \boldsymbol{t} \right)\exp\left( \frac{\boldsymbol{Total ponts}}{\boldsymbol{10}} \right)$. The baseline survival, denoted as s0(t), varies depending on the specific characteristics of the individual. As an example, consider a male resident who is 86 years old and has been diagnosed with Parkinson's disease, dementia, cancer, hypertension, heart failure and arthritis. This individual has a history of falls within the three months prior to entering RACFs but has not experienced any falls since admission. Currently in his tenth month at the facility, he is taking six regular medications, which include an anti-Parkinson's drug, diuretics, opioids, calcium channel blockers and drugs for the treatment of bone disease. Considering these relevant factors, this individual accumulates a total of eight points in the risk assessment (i.e., four points for falls history, four points for the anti-Parkinson's drug, two points for opioids and a deduction of two points for the bone medication). As a result, his estimated risk of falling in the tenth landmark month is 7.6%. |
| --- |
